# Quorum Sensing System Affects the Plant Growth Promotion Traits of *Serratia fonticola* GS2

**DOI:** 10.3389/fmicb.2020.536865

**Published:** 2020-10-30

**Authors:** Byung Kwon Jung, Jerald Conrad Ibal, Huy Quang Pham, Min-Chul Kim, Gun-Seok Park, Sung-Jun Hong, Hyung Woo Jo, Chang Eon Park, Seung-Dae Choi, Yeongyun Jung, Setu Bazie Tagele, Jae-Ho Shin

**Affiliations:** ^1^School of Applied Biosciences, College of Agriculture and Life Sciences, Kyungpook National University, Daegu, South Korea; ^2^CJ Blossom Park, Suwon-si, South Korea; ^3^Atogen Co., Ltd., Daejeon, South Korea; ^4^Yeongnam Regional Office, Animal and Plant Quarantine Agency, Busan, South Korea; ^5^R&I Center, COSMAX BTI, Seongnam, South Korea

**Keywords:** ACC deaminase, biofilm, indole acetic acid, plant growth promotion, quorum sensing

## Abstract

Quorum sensing (QS) enables bacteria to organize gene expression programs, thereby coordinating collective behaviors. It involves the production, release, and population-wide detection of extracellular signaling molecules. The cellular processes regulated by QS in bacteria are diverse and may be used in mutualistic coordination or in response to changing environmental conditions. Here, we focused on the influence of the QS-dependent genes of our model bacterial strain *Serratia fonticola* GS2 on potential plant growth promoting (PGP) activities including indole-3-acetic acid (IAA) production, 1-aminocyclopropane-1-carboxylate (ACC) deaminase activity, and biofilm formation. Based on genomic and phenotypic experimental data we identified and investigated the function of QS genes in the genome of the model strain. Our gene deletion study confirmed the biological functionality of the QS auto-inducer (*gloI*) and receptor (*gloR*) on potential PGP activities of GS2. A transcriptomic approach was also undertaken to understand the role of QS genes in regulation of genes primarily involved in PGP activities (IAA, ACC deaminase activity, and biofilm formation). Both transcriptomic and phenotypic data revealed that the QS-deletion mutants had considerably less PGP activities, as compared to the wild type. In addition, *in vivo* plant experiments showed that plants treated with GS2 had significantly higher growth rates than plants treated with the QS-deletion mutants. Overall, our results showed how QS-dependent genes regulate the potential PGP activities of GS2. This information may be helpful in understanding the relationship between QS-dependent genes and the PGP activity of bacteria, which aid in the production of practical bio-fertilizers for plant growth promotion.

## Introduction

Quorum sensing (QS) is a process by which bacteria coordinately regulate gene expression in response to sensing of diffusible chemical signals (Kleerebezem et al., 1997; Miller and Bassler, 2001). QS involves the production, release, and population-wide detection of extracellular signaling molecules, which are called autoinducers. The concentration of autoinducers increase in the environment as bacterial population density increases (Papenfort and Bassler, 2016). Preliminary research on *N*-acyl homoserine lactone (AHL)-based QS began in the 1960s when it was found that liquid cultures of the marine bioluminescent bacteria *Vibrio fischeri* produced light only when large numbers of bacteria were present (Greenberg, 1997; Parsek and Greenberg, 2000). QS makes use of different types of signaling systems, including autoinducer-1 (AI-1), which is widely conserved across phylogenetically diverse bacterial species, and autoinducer-2 (AI-2). Furthermore, signaling compounds include *Pseudomonas* quinolone signal (PQS), diffusible signal factor (DSF), and autoinducer-3 (AI-3) (LaSarre and Federle, 2013).

Gram-positive and Gram-negative bacteria use different types of QS systems. Gram-positive bacteria use autoinducing peptides (AIP) as signaling molecules while the most common class of autoinducers in Gram-negative bacteria are AHLs, which contain a primary *N*-acylated homoserine lactone ring and a 4–18 carbon acyl chains that contain variations. The length of the acyl chain may affect the stability of the molecules, which may further influence signaling dynamics. Many bacterial species contain LuxI-type synthases that produce these AHLs (Whitehead et al., 2001; Ng and Bassler, 2009; Rutherford and Bassler, 2012; Papenfort and Bassler, 2016). The LuxI homolog is an AI synthase that catalyzes the reaction between an acyl carrier protein (ACP) and *S*-adenosylmethionine (SAM) for production of AHLs. The AHLs then bind to cytoplasmic LuxR-like transcription factors, resulting in modulation of target gene regulation.

Cellular processes regulated by QS in bacteria are diverse and include the production of signal molecules. QS molecules and identical two-component regulatory systems are used by plant-interacting bacteria either in coordinating mutualistic or pathogenic associations or in response to changing environmental conditions (Soto et al., 2006). QS molecules may bring together major traits exhibited by rhizosphere bacteria that are essential for plant growth promotion. In a recent study, Zúñiga et al. (2017) reported that QS systems are vital in host colonization and establishment by *Burkholderiales* species. Similarly, the role of AHL and their analogs in the symbiotic interactions of *Rhizobium*-legume and *Pseudomonas fluorescens* on root colonization has been previously reported (Wei and Zhang, 2006; Zúñiga et al., 2017). Müller and colleagues reported on the influence of QS on the biocontrol activity of *Serratia plymuthica* (Müller et al., 2009). Similarly, Ortiz-Castro and Lopez-Bucio reviewed plant responses to a variety of AHLs produced in the rhizosphere, including immune and defense responses and root growth and development (Ortiz-Castro et al., 2009).

A variety of AHLs and their production mechanisms have been described in a number of *Serratia* spp., which demonstrate the specificity and diversity of QS signal molecules in regulation (Wei and Zhang, 2006). The genus *Serratia* has been found to inhabit an array of environments, including water, plants, soil, and mammals (Grimont and Grimont, 2006). AHL and AI-2 dependent QS systems have been described in the *Serratia* species (Christensen et al., 2003; Van Houdt et al., 2007).

Currently, many QS studies have focused on pathogenic bacteria, which are of minor use for the agricultural industry. Moreover, the QS-regulated beneficial properties in plant growth-promoting rhizobacteria (PGPR) as well as arbuscular mycorrhizal fungi (AMF) have not been fully exploited. PGPR are the most effective and best studied soil microorganisms which can promote plant growth performance. Different mechanisms are used by PGPR to enhance plant growth. These PGPR produce various compounds such as phytohormones, organic acids, and siderophores. They are also involved in fixing atmospheric nitrogen, solubilizing phosphate and producing antibiotics which suppress deleterious rhizobacteria (Jha and Saraf, 2015).

A previous study highlighted the QS properties and the potential plant growth-promoting (PGP) activity of *Serratia fonticola* GS2 (Jung et al., 2017). Here, we investigated the function of AHL-mediated QS in regulating the potential PGP activities of *S. fonticola* GS2, including indole-3 acetic acid (IAA) production, 1-aminocyclopropane-1-carboxylic acid (ACC) deaminase, biofilm formation, and volatile compound production through genomic, transcriptomic, and phenotypic analysis. A genomic approach was done to investigate the QS-dependent genes. The identification of the genes allowed us to make deletion mutants which we then compared the gene expression through transcriptomics, and we further confirmed the effect of the mutants on PGP activities through a phenotypic analysis.

## Materials and Methods

### Identification of Putative LuxI/R-Type Quorum Sensing Dependent Genes

Predicted open reading frames (ORFs) were further annotated by comparing the sequences against NCBI-NR^1^ and Uniprot databases^2^ to identify the genes *gloI* and *gloR*, encoding AHL synthase and an AHL receptor protein, respectively. The predicted protein of *gloI*/*R* was then queried against the NCBI conserved domain database (Marchler-Bauer et al., 2015) to validate the authenticity of the putative QS genes. The putative translation product of *gloI*/*R* was used to conduct a BLAST search for non-redundant protein sequences (nr) in the NCBI database^3^; homologous sequences were selected as references. Furthermore, phylogenetic trees of putative *gloI* and *gloR* sequences were constructed using MEGA6 software. The ClustalW program was employed for multiple alignment of the sequences. The neighbor-joining method with 1,000 bootstrap replications was used to generate the phylogenetic trees. The following reference sequences were used to construct the phylogenetic trees (accession number): YpeI, *Serratia glossinae* (WP_021807856.1); LuxI, *Serratia plymuthica* (WP_062869424.1); ExpI, *Erwinia piriflorinigrans* (WP_023655885.1); LuxI, *Erwinia tasmaniensis* (WP_012440701.1); LuxI, *Serratia marcescens* (EMF05894.1); LuxI, *Trabulsiella odontotermitis* (WP_054180300.1); LuxI, *Trabulsiella guamensis* (WP_038163062.1); LuxI, *Enterobacter lignolyticus* (WP_013366030.1); LuxI, *Klebsiella* sp. (WP_062741329.1); LuxI, *Pectobacterium carotovorum* subsp. *carotovorum* (WP_040032263.1); YspR, *Serratia glossinae* (WP_021807855.1); LuxR, *Erwinia tasmaniensis* (WP_012440700.1); ExpR, *Erwinia piriflorinigrans* (WP_023655886.1); EsaR, *Serratia plymuthica* (WP_065505835.1); LuxR, *Trabulsiella guamensis* (KFB98059.1); LuxR *Serratia marcescens* (WP_049198804.1); LuxR, *Klebsiella oxytoca* (WP_064290786.1); LuxR, *Serratia ureilytica* (WP_046688133.1); LuxR, *Trabulsiella odontotermitis* (WP_049847891.1); LuxR, *Enterobacter lignolyticus* (WP_013366031.1) (Supplementary Figure 1).

### Mutagenesis of QS-Dependent Genes in *Serratia fonticola* GS2

To construct the Δ*gloI* (AI) and Δ*gloR* (RT) mutants, we employed a strategy in which the gene of interest was deleted from the chromosome while leaving the start and stop codons intact. For this, we PCR-amplified two fragments of DNA flanking the gene of interest: fragment A (150 bp), located upstream of *gloI* and *gloR*, and fragment B (150 bp), located downstream of *gloI* and *gloR*. For amplified *gloI*, fragment A was amplified using primer gloI30-1 containing a *Pst*I restriction site and primer gloI188-3. Fragment B was amplified using primer gloI618-2 containing a *Sac*I restriction site and primer gloI469-4. For amplified *gloR*, fragment A was amplified using primer gloR33-1 containing a *Pst*I restriction site and primer gloR183-3. Fragment B was amplified using primer gloR684-2 containing a *Sac*I restriction site and primer gloR534-4. Primers gloI188-3, gloI469-4 and gloR183-3, gloR534-4 are complementary, respectively, and therefore, fragments A and B can be fused in a second round of primerless PCR to allow the polymerase to anneal fragments A and B in a strand reaction under the following conditions: 5 min hot start activation followed by 10 cycles of 94°C (30 s), 55°C (30 s), and 72°C (50 s). A final PCR using primer-1 and primer-2 was then conducted to enrich for the full-length 0.3 kb fusion product of fragments A and B. This fragment was digested with the enzymes *Pst*I and *Sac*I and ligated into the already digested suicide plasmid pDS132, resulting in pDS132ΔgloI and pDS132ΔgloR. Plasmids pDS132ΔgloI and pDS132ΔgloR were inserted into *E. coli* BW20767 by electroporation and conjugated into ampicillin-resistant strain GS2.

For conjugation, donor strains *E. coli* BW20767 (pDS132ΔgloI) and *E. coli* BW20767 (pDS132ΔgloR) and the recipient strain *S. fonticola* GS2 were grown in Lysogeny Broth (LB) (Tryptone 10 g, yeast extract 5 g, and NaCl 10 g in 1 L of water) broth until late log phase (OD600 nm = 0.8). Cells were then mixed at an equal volume and spotted onto nylon filter paper on an LB agar plate. After 12 h of conjugation at 30°C, the cells were recovered by washing the filters with fresh LB broth, followed by plating on LB agar containing ampicillin (50 μg/mL) and chloramphenicol (10 μg/mL). After 16 h of incubation at 30°C, a colony was picked and suspended in fresh LB broth, and serial dilutions were plated on LB agar containing 5% sucrose and no NaCl. After overnight incubation at 30°C, 100 colonies were streaked on chloramphenicol-containing LB agar and LB agar with 5% sucrose and no NaCl. Sucrose-resistant and chloramphenicol-sensitive clones were confirmed by PCR using plasmid-specific primers pDS132-specific-F and pDS132-specific-R (Supplementary Table 1).

For the gene deletion complementation, full-length *gloI* and *gloR* genes were amplified by PCR from *S. fonticola* strain GS2 chromosomal DNA using the primers gloIF-gloIR and gloRF-gloRR with restriction enzyme linkers (*Nhe*I and *Xba*I) (Supplementary Table 1). The amplified genes were digested with *Nhe*I and *Xba*I and ligated into the broad-host-range expression vector pBTBXh-3, which had been digested with the same enzymes. The plasmids pBTBXh-gloI and pBTBXh-gloR, containing the full length *gloI* and *gloR* genes, respectively, were propagated in *E. coli* DH5α and introduced into the gene-deleted mutant strains termed AI and RT, respectively.

A bioassay was conducted using the deletion mutants, AI and RT, and wild type (WT) GS2. Autoinducer bioassay (AB) minimal medium containing 0.2% mannitol, phosphate buffer, trace elements, and X-gal (60 μg) was used for growth of the AHL indicator strain *A. tumefaciens*. For growth complemented mutant strains, LB medium was supplemented with 0.35% L-arabinose. Antibiotics were used in the selection media at the following concentrations: *E. coli*, ampicillin (100 μg/mL) and chloramphenicol (30 μg/mL); *A. tumefaciens*, gentamicin (15 μg/mL); and *S. fonticola*, ampicillin (50 μg/mL) and chloramphenicol (10 μg/mL). LB agar with 5% sucrose and no NaCl was used to select for plasmid excision from the chromosome during the gene allelic exchange experiments. A blue color which results from hydrolysis of X-gal through the expression of the β-galactosidase gene indicates production of AHL. The bacterial strains and plasmids used in this study are listed in Supplementary Table 2.

### RNA Extraction and Transcriptome Analysis of the WT and Mutant Strains

Triplicates of GS2 WT and mutant strains were grown in LB medium at 30°C for 20 h, and the cells were harvested by centrifugation at room temperature and washed with phosphate buffer (pH 7.0). Total RNA was extracted using a modified method with a FastRNA Pro Blue Kit (MPBiodmedicals, Santa Anna, CA) and TRI reagent^®^ (Sigma-Aldrich, St. Louis, MO). The extracted total RNA from the three samples was sent to DNA Link (Seoul, South Korea)^4^. Another set of sample replicates were sent for enrichment of mRNA and subsequent sequencing.

Raw sequence files were quality checked. The high quality reads were aligned to the reference genome of *Serratia fonticola* GS2 (NZ_CP013913.1) through HISAT2 using the default parameters. The reads were sorted using SAMtools. The sorted bam files were used to count the genes using the program featureCounts (Liao et al., 2014; Kim et al., 2019). The read count per gene measurements for each replicate were summed across sequencing lanes to give read count per gene for each of the samples. The gene counts were then normalized using DESeq2 and the ViDGER package in R (McDermaid et al., 2018).

### *In vitro* Assay for Potential Plant Growth-Promoting Traits

In order to examine growth curves of *S. fonticola* GS2, fresh broth cultures of the WT and mutant strains were inoculated into 100 mL of LB medium and incubated at 30°C in a shaking incubator at 200 rpm. The cell density was determined by enumerating bacterial cells every 2 h within 12 h of incubation and at an interval of 12 h from 12 to 168 h of incubation.

Total indolic compounds produced by the strains were quantified using Salkowski reagent (50 mL 35% perchloric acid, 1 mL 0.5 FeCl_3_). The absorbance of the pink indole complex was measured at 535 nm. The calibration curve was constructed using indole-3-acetic acid (IAA) standards (Sigma-Aldrich, St. Louis, MO). The production of IAA in the culture media was confirmed by gas chromatography-mass spectrometry equipped with selected ion monitoring (GC-MS-SIM).

The activity of 1-aminocyclopropane-1-carboxylic acid (ACC) deaminase was assessed as described previously (Jacobson et al., 1994). Briefly, WT and mutant strains were grown in LB medium at 30°C for 24 h. The cells were collected by centrifugation, and the pellets were washed twice using 0.2 M phosphate buffer (pH 7.0). The cells were then re-suspended in Dworkin and Foster salt minimal medium with 3 mM ACC as the sole nitrogen source. ACC deaminase activity was measured by determining the concentration of α-ketobutyrate at 540 nm. A colormetric assay of biofilm formation with crystal violet staining was performed as previously described (Jackson et al., 2002).

Voges-Proskauer (VP) test was employed to detect acetoin production by wild-type and mutant strains. The strains were grown in MR-VP medium (0.35% pancreatic digest of casein, 0.35% peptic digest of animal tissue, 0.5% potassium phosphate, 0.5% dextrose) at 30°C for 48 h. We added 0.5 mL of α-naphthol (5%, w/v ethanol) and 0.5 mL of potassium hydroxide (40%, w/v water) into 2 mL of culture broth. The resulting mixture was incubated for 30 min at the room temperature. A red ring on the surface of the culture was considered positive for acetoin.

### *In vivo* Pot Trial

A pot test with the rice cultivar Waito-c (*Oryza sativa* L.) was carried out to determine the effect of bacterial strain GS2 on plant growth, morphology, and physiology. Seeds were surface sterilized with 70% ethanol for 5 min and disinfected using a 3:2:2 (v/v/v) ratio of Clorox:distilled water:0.05% Triton X-100 for 1 min, followed by rinsing four times in sterile distilled water. The sterilized seeds were then vernalized in a refrigerator at 4°C for 72 h, placed on sterile wet filter papers in Petri-dishes, and germinated for 48 h in a growth chamber. Four rice seedlings were each transplanted into a pot filled with 50 g of sterilized soil. The plants were maintained in a growth chamber with a day:night cycle of 16 h:8 h (20,000 lux light intensity) and temperatures of 26:20°C, respectively, with 65% relative humidity. Meanwhile, strain GS2 was incubated at 30°C for 72 h at 200 rpm. The cells were pelleted, re-suspended in 50 mL sterile distilled water, and inoculated at 10^8^ CFU/mL per pot. After 3 weeks of treatment, growth attributes such as plant length, chlorophyll content, and fresh weight were recorded, and the inoculation effect was compared with distilled water-treated control seedlings. The experiment consisted of 10 plants per treatment and was performed in triplicate.

### Plant-Bacteria Dual Growth Experiment and Gas Chromatography-Mass Spectrometry (GC-MS) Analysis of Microbial Volatile Compounds

*Arabidopsis thaliana* Col-0 was used for plant-bacteria dual growth experiments. Seeds were sterilized by vortexing in 1 mL of 70% ethanol in 1.5 mL tubes for 5 min. The supernatant was removed, and 1 mL of disinfectant (Clorox:distilled water:0.05% Triton X-100 in a v/v/v ratio of 3:2:2) was added, and the tubes were strongly shaken for 10 min. The seeds were washed five times with sterile distilled water after centrifugation and removal of the supernatant. The sterilized seeds were then vernalized at 4°C for 3 days. To prepare seedlings, the seeds were placed on a square Petri-dish containing half-strength Murashige-Skoog medium (MS) (0.22 g of micro and macro nutrients including vitamins (glycine, myo-inositol, nicotinic acid, pyridoxine HCl, and Thiamine HCl) in 100 mL SDW), and the plates were sealed with parafilm and incubated in the dark for 6 days at 20°C and 60% relative humidity.

Dual growth experiments were performed in two-compartment petri dishes, wherein 6-day old seedlings (*n* = 16) were placed in one half of the compartment containing half-strength MS agar, and the other half compartment contained Methyl Red Voges-Proskauer MR-VP (0.35% pancreatic digest of casein, 0.35% peptic digest of animal tissue, 0.5% potassium phosphate, 0.5% dextrose) and LB media. The MR-VP and LB containing compartments were inoculated with different concentrations of one, three and nine drops of 10 μL (10^9^ CFU/mL) of each strain. A sterile MR-VP and LB media without bacteria inoculation served as controls. The compartment plates were sealed with parafilm and incubated in a plant growth chamber with a 12 h day: 12 h night cycle at 20°C and 60% relative humidity for 2 weeks. Experiments were conducted in triplicate. The results were expressed as a percentage of plant biomass (fresh weight) relative to the biomass of the control plants.

Volatile compounds released by WT and mutant strains were analyzed using GC-MS. Briefly, all strains were cultured in 1 L each of LB and MR-VP broth for 48 h at 30°C. The supernatant was harvested by centrifugation for 30 min at 6,000 × g and 4°C and filtered through a 0.45 μm pore cellulose acetate filter. The filtered supernatant was extracted three times with two volumes of ethyl acetate, and the organic solvent phase was evaporated using a rotary evaporator. The extract was re-dissolved in 1 mL of methanol and analyzed by GC-MS (6890N Network GC System, Agilent Technologies, Santa Clara, CA).

## Results

### Identification and Mutagenesis of Quorum Sensing Dependent Genes in *S. fonticola* GS2

*Serratia fonticola* GS2 was previously studied and found to exhibit QS and QS signaling AHLs molecules, *N*-hexaonyl-_L_-homoserine lactone (HHL) and *N*-octanoyl-_L_-homoserine lactone, were detected via thin layer chromatography (TLC) and high performance liquid chromatography (HPLC) (Jung et al., 2017). In the present study, we located the putative *luxI* and *luxR* homologs in the annotated GS2 genome. The phylogenetic analysis revealed that a predicted protein coded by a putative 645 bp *N*-acyl homoserine lactone synthase (*gloI*) gene had high amino acid sequence similarity (99%) with the LuxI homolog of *S. fonticola* (WP 021807856.1). Conserved domain analysis of the predicted protein product of this gene revealed the presence of an autoinducer synthase domain (Pfam accession number: PF00765), further confirming that the gene found in GS2 is an authentic LuxI homolog. A 717 bp putative QS transcriptional activator gene (*gloR*) exhibited the highest sequence similarity (99%) with the LuxR homolog of *S. fonticola* (WP_021807855.1). The putative LuxR homolog was deemed to be authentic. The predicted protein sequence was analyzed and confirmed to contain the universal conserved domain organization of the autoinducer binding domain (Pfam accession number: pfam03472). The *gloR* gene of GS2 was also found to be in a convergent transcriptional orientation relative to the *gloI* gene. The close proximity of the LuxR homolog to the LuxI homolog is commonly observed in a typical LuxI/LuxR-type QS circuit (Schaefer et al., 2013). However, the LuxI and LuxR homologs of the closely related species, namely *Serratia* and *Erwinia* which are classified under the family Yersiniaceae, and Erwiniaceae, respectively, were grouped in a different clade (Supplementary Figures 1A,B). After identification of the LuxI/LuxR-type genes and to further study the QS mechanisms of GS2, the *gloI* and *gloR* genes were deleted. PCR analysis of the AI mutant and RT mutant DNA produced the expected amplicon size of 300 bp (Supplementary Figure 2A). The *gloI* and *gloR* genes encode *N*-acyl homoserine lactone (AHL) synthase and the quorum-sensing transcriptional activator that is required for production of AHL. Subsequently, *gloI* and *gloR* mutant strains failed to induce AHL production in *A. tumifaciens*. Interestingly, AHL production in *gloI* and *gloR-*deleted mutants was restored by the complementation of the respective genes, pBTBXh-gloI and pBTBXh-gloR plasmids, respectively (Supplementary Figure 2B).

### Transcriptome Analysis of the Two Mutant Strains and Wild-Type

The identification of an intact *N*-acyl homoserine lactone (AHL) quorum-sensing system in *S. fonticola* strain GS2 suggested the presence of AHL-regulated functions. To gain insight into the possible genes regulated by QS in GS2, we performed transcriptional profiling of the WT strain and the two mutants. We obtained average reads of 29,177,283; 22,233,487; and 32,289,539 for WT, AI, and RT, respectively. The reads were then aligned using HISAT2 obtaining average percent mapped reads of 94, 78.31, and 87.90 for WT, AI, and RT, respectively. The gene counts were obtained using featureCounts and subsequently normalized using DESeq2, which estimates the mean-variance dependence, and differentially expressed genes (DEGs) were used for further downstream analysis.

We detected a total of 142 DEGs (*p* < 0.05) in both mutants. It was observed that most of the genes were downregulated (Supplementary File). Out of 142 DEGs, only two genes viz., aldo/keto reductase and cysteine desulfurase *SufS* were observed to be shared as being DEGs in both mutants. In AI mutant, aldo/keto gene was upregulated while the 103 DEGs were down regulated. On the other hand, out of the 40 detected DEGs in the RT mutant, two genes, namely the aldo/keto gene and the AEC family transporter were upregulated (Additional File).

**FIGURE 1:**
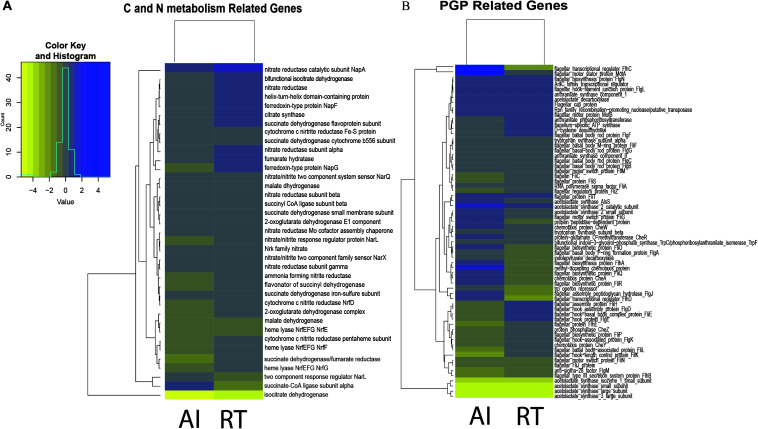
**(A)** Heatmap representation of differentially expressed genes involved in plant growth promotion by *Serratia fonticola* GS2. The genes involved in plant growth promotion (tryptophan biosynthesis, ACC deaminase production, acetoin production, and biofilm synthesis). **(B)** Heatmap representation of differentially expressed genes involved in carbon and nitrogen metabolism in *Serratia fonticola* GS2. The genes involved in plant growth promotion (TCA cycle and nitrogen metabolism. The genes were based on the whole genome, mapped to KEGG. The yellow color shows down regulated genes while the blue color represents the up regulated genes). Where AI, autoinducer-deleted mutant, RT, receptor-deleted mutant were compared against the WT: control.

In this study we aimed to determine if there is a relationship between QS genes and PGP activities. Thus, we focused our analysis on genes involved in PGP activities. Comparison of the gene expression for both the autoinducer-deleted and receptor-deleted mutants against the wild type are shown in Figure 1A. Genes involved in PGP activities such as auxin, acetoin, ACC deaminase, and biofilm production were downregulated, though statistically non-significant, in the mutants compared to wild type. Up regulation of *trpA*, *trpB*, *trpC/F*, and *trpE* genes, while marginal occurred in both the AI and RT mutants, genes which are involved in the biosynthesis of tryptophan, the precursor of IAA. The *trpD* and *trpG* genes showed down regulation in both the mutants. We observed that the RT mutant (four out of the seven genes were down regulated) was more affected in contrast with AI (two out of seven showed a negative fold change) in terms of genes involved in IAA production. In a similar manner, *aldB, budB, ilyB* genes involved in acetoin production, were found to be minutely regulated in both mutants but *ilyH* was shown to have at least a twice the negative fold change for both mutants. Although the ACC deaminase gene was not detected in the transcriptome result perhaps as a result of annotation error, but we confirmed through BLAST result that the *acds* gene is present in the same domain as the D-cysteine desulfhydrase. A previous study by Nascimento et al. (2014) discussed that nucleotide sequences coding for D-cysteine desulfhydrases are considered as ACC deaminases. D-cysteine desulfhydrase was found to be down regulated in both the AI and RT mutants. Meanwhile, most of the biofilm production-related genes, those which are also involved in motility and flagellar assembly, were down regulated in both the AI and RT mutants. More than half of the observed gene showed a negative fold change. Of the 46 genes we observed, 25 showed a negative fold change for RT while 24 genes were observed to be down regulated in the AI mutant.

Moreover, we wanted to see if there was an indirect effect of the low production of PGP activities through carbon and nitrogen metabolism (Figure 1A). Since the QS gene is considered as a global regulator, we determined whether gene deletion affected carbon and nitrogen metabolism (Sperandio et al., 2001, 2002). We observed that the genes involved in the TCA cycle for both the mutants were down regulated as well, with a few exceptions having nominal regulation. However, for the case of nitrogen metabolism, the AI mutant (15 out of the 23 observed genes) showed considerably more down regulated genes in comparison to the RT (6 of the 23 observed genes) mutant, as seen in Figure 1B.

### Growth Characteristics and *in vitro* Plant Growth-Promoting (PGP) Traits of the Strains

**FIGURE 2:**
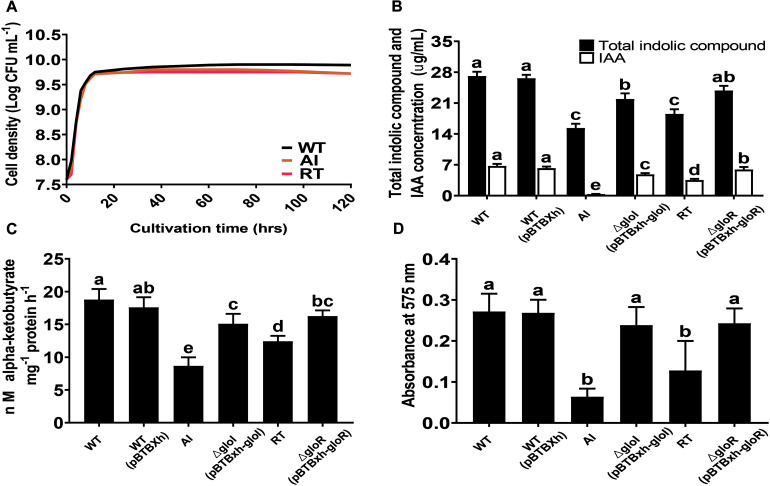
Plant growth-promoting related phenotype analysis. **(A)** Growth curves are shown for *Serratia fonticola* GS2 wild-type (black), AI mutant (orange), and RT (red) mutant. All strains were grown in LB medium at 30°C. **(B)** Quantification of total indolic compounds and indole-3-acetic acid (IAA). **(C)** 1-aminocyclopropane-1-carboxylic acid production based on α-ketobutyrate production. **(D)** Biofilm formation. Error bars expressed means of standard error (SE). Values with different letters are significantly different according to Duncan’s multiple range test (DMRT), *p* < 0.05.

The growth characteristics of GS2-WT, AI, and RT were studied using LB media at 30°C. The results showed that all strains grew well and had similar growth rates during their exponential phase. However, during the transition to stationary phase, the cell density of the mutant strains was lower than that of the WT strain (Figure 2A). *S. fonticola* GS2 was used to investigate *in vitro* PGP traits that included IAA, ACC deaminase, and biofilm production. Salkowski test and GC-MS analysis were performed to determine the ability of the GS2 WT, AI, and RT strains to produce indolic compounds and IAA (Supplementary Figure 3). Total indolic compounds and IAA production by the mutant strains were significantly decreased, in comparison to the WT strain. Indolic compounds and IAA production were successfully restored in complemented strains *C-gloI* and C-*gloR* (Figure 2B). The mutant strains exhibited significantly lower ACC deaminase activity than the WT and complemented strains, indicating that ACC deaminase activity was restored by complementation (Figure 2C). The WT, mutant, and complemented strains were stained with crystal violet to examine biofilm forming abilities. Deletion mutants AI and RT showed a decrease in biofilm formation during the 48 h of incubation, while the difference in formation between complemented strains and the WT was insignificant (Figure 2D), indicating biofilm forming ability was restored in the complemented strains.

To further investigate acetoin production in the WT and mutant strains, we used MR-VP medium. Our qualitative data showed that all the strains were able to produce acetoin exhibiting a red ring on the surface of the culture (Supplementary Figure 4). Similarly, AI and RT mutants produced lower amount of ACC deaminase and IAA in comparison to the wild-type. Thus, the repressed PGP traits in AI and RT suggest that *gloI* and *gloR* could be responsible in regulating the production of the stated PGP traits in *S. fonticola*.

### *In vivo* Pot Trial and Plant–Bacteria Dual Growth Experiment

The effect of AI and RT on plant growth promotion activity was evaluated using a pot trial with the gibberellin-deficient rice mutant Waito-c. Growth parameters were measured and recorded for 3 weeks after planting (Table 1). Results showed that inoculation with *S. fonticola* GS2 had significant effects on various attributes of rice growth, in comparison to the AI and RT strains (Table 1). Plants inoculated with complemented strains displayed a positive growth effect similar to GS2, as determined by root length. The mutant strains resulted in significantly (*p* < 0.05) decreased root lengths and chlorophyll content, as well as fresh and dry biomass content (Table 1).

**TABLE 1:** Effect of *Serratia fonticola* GS2 wild-type and mutant strains on plant growth attributes and chlorophyll content on rice cultivar Waito-c (*Oryza sativa* L.).

Treatments	Shoot length (cm)	Root length (cm)	Chlorophyll content (SPAD)	Fresh biomass (g/plant)	Dry biomass (mg/plant)
Water only	20.4 ± 0.4^ab^	6.6 ± 0.3^d^	37.0 ± 0.4^c^	1.7 ± 0.1^cd^	75.9 ± 1.1^c^
WT	20.9 ± 0.2^a^	11.0 ± 0.7^a^	44.8 ± 0.3^a^	2.0 ± 0.1^a^	98.4 ± 1.4^a^
WT (pBTBXh)	20.7 ± 0.2^ab^	10.5 ± 0.3^ab^	42.8 ± 1.0^ab^	1.9 ± 0.1^ab^	93.3 ± 1.2^ab^
AI	20.1 ± 0.5^ab^	7.6 ± 0.5^cd^	37.2 ± 1.5^c^	1.7 ± 0.1^cd^	74.3 ± 1.5^c^
*C-gloI* (pBTBXh-gloI)	20.5 ± 0.1^ab^	9.5 ± 0.4^ab^	41.4 ± 0.2^b^	1.8 ± 0.1^ab^	91.7 ± 1.5^b^
RT	19.9 ± 0.2^b^	7.4 ± 0.4^d^	37.0 ± 1.7^c^	1.7 ± 0.1^d^	75.5 ± 1.4^c^
*C-gloR* (pBTBXh-gloR)	20.2 ± 0.4^ab^	9.0 ± 0.7^bc^	41.6 ± 0.4^b^	1.8 ± 0.1^bc^	90.2 ± 4.0^b^

*Values are expressed as the means ± SE. Different letters in columns are significantly different according to Duncan’s multiple range post-hoc tests (p < 0.05).*

**FIGURE 3:**
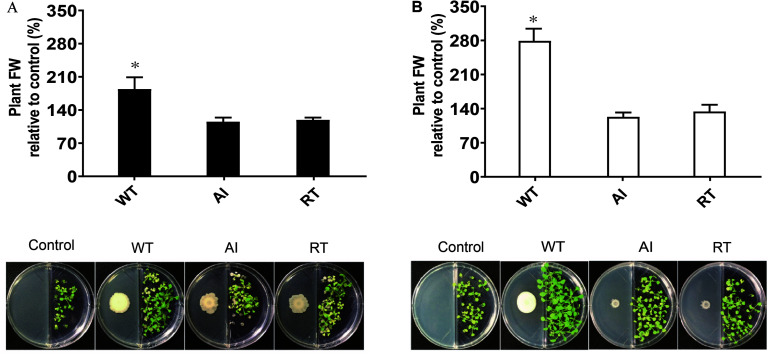
The effect of the volatile compounds of *Serratia fonticola* strain GS2 wild-type and mutants on the growth of *Arabidopsis thaliana* Col-0. **(A)** Volatile effect of the strains on the fresh weight of *A. thaliana* Col-0 relative to the control and a pictorial representation of *A. thaliana* Col-0 with bacteria cultivated on LB and **(B)** MR-VP. Error bars represent standard errors, and results marked with an asterisk (*) are significantly different from the non-inoculated control according to a Student’s *t*-test (*p* < 0.05, *n* = 48).

Additionally, the effect of volatile compounds emitted from all strains grown in LB and MR-VP media was tested on the growth of *A. thaliana* Col-0 (Figure 3). The plants were harvested after 2 weeks and the fresh weight was determined to estimate biomass content. The results showed that plants exposed to volatiles emitted by GS2 had a higher biomass content, as compared to plants exposed to volatiles produced by the mutant strains at all concentrations (Supplementary Figure 5). Moreover, as the concentrations of each stains was increased the growth of *A. thaliana* Col-0 was reduced, which might be due to the emission of inhibitory volatile compounds (Supplementary Table 3). Hence, in order to investigate the volatile compounds which could potentially either promote or inhibit the growth of plants, profiling of volatiles was performed using GC-MS analysis. The GC-MS data revealed that among the 20 volatile compounds detected, 18 of them are known to have plant growth effect (Table 2). The emission of acetoin, 3-(methylthio)-1-propanol, tryptophol, and octadecanoic acid, which have plant growth stimulating activities, were highly reduced in the mutant strains, as compared to WT. Plant growth inhibitors of cyanide compounds including methyl o-cyanobenzoate, 1,1-dicyano-2phenylethylene, and phenylcyanamide were detected in all strains, particularly in LB media.

**TABLE 2:** The production of volatile organic compounds by *Serratia fonticola* strain GS2 wild-type and mutants as measured by GC-MS.

Compound	Function	R/T (min)	Peak area (%)					
			LB			MR-VP		
			WT	AI	RT	WT	AI	RT
Acetoin	Plant growth stimulator	3.88	nd	nd	nd	4.71	2.70	0.97
Benzaldehyde	Plant growth regulator	7.30	0.28	0.15	0.25	1.17	0.20	0.42
3-(Methylthio)-1-propanol	Plant growth regulator	7.89	2.93	1.60	1.69	nd	nd	nd
S-Methyl methanethiosulfinate	Antimicrobial activity	8.01	0.30	0.25	0.19	nd	nd	nd
Benzeneacetic acid	Natural auxin	27.02	nd	nd	nd	2.87	0.94	0.26
9-Borabicyclo[3.3.1]nonan-9-ol	Boron deficiency defense	36.38	nd	nd	nd	0.39	nd	nd
Dodecanoic acid	Plant growth regulator	36.68	nd	nd	nd	1.44	nd	nd
2-(Dimethylhydrazone)butanal	Plant growth regulator	38.08	nd	nd	nd	1.20	3.71	2.22
2-methyl-3-furanthiol	Plant growth regulator	38.43	nd	nd	nd	0.29	nd	nd
Tryptophol	Natural auxin	38.53	3.64	nd	nd	2.32	nd	nd
Methyl o-cyanobenzoate	Plant growth inhibitor	38.76	2.53	1.87	0.76	nd	nd	nd
Isopropyl S-2-(diisopropylamino)ethyl isopropylphosphonothiolate	–	39.01	nd	nd	nd	0.64	nd	nd
1,1-Dicyano-2-phenylethylene	Plant growth inhibitor	39.08	nd	nd	nd	nd	2.3	3.41
1-Tetradecanol	Plant growth regulator	39.39	nd	nd	nd	0.36	nd	nd
Hydantoin, 1-butyl	Plant growth regulator	39.51	nd	nd	nd	0.27	nd	nd
(E)-4-Nitrophenylazo tert-butyl sulfide	–	40.04	nd	nd	nd	1.29	nd	nd
Phenylcyanamide	Plant growth inhibitor	41.16	nd	0.23	0.28	nd	nd	nd
Octadecanoic acid	Plant growth stimulator	41.33	0.12	nd	nd	0.87	0.30	0.12
2,5-Piperazinedione, 3-(phenylmethyl)-	Plant growth regulator	41.82	0.15	0.11	0.14	1.77	0.97	0.54
2,5-Difluorobenzoic acid	Plant growth regulator	44.06	nd	nd	nd	0.54	nd	nd

*Where: R/T is the retention time and nd is not detected.*

## Discussion

In this study, we assessed the effect of the LuxI-R QS system on PGP activities. We used *Serratia fonticola* GS2 as a model organism, as it possesses both QS and potential PGP activities. We first located the putative *luxI* and *luxR* homologs in the annotated genome. Then, the predicted protein product of *gloI*/*R* was queried against the NCBI conserved domain database for validation of the authenticity of the putative QS genes. To find out about the relationship of the QS genes with PGP activities, deletion of *gloI* and *gloR* was carried out and confirmed through PCR. The WT, RT, and AI mutants were compared by carrying out different approaches. First, transcriptome analysis was used to gain insight into the role of AHL QS on the potential PGP qualities of GS2 by comparing the DEGs between the WT and the two mutant strains. We initially looked at genes involved in production of auxin, ACC deaminase, acetoin, and biofilms, characteristics that contribute to PGP activity. The PGP traits were tested *in vitro* using the Salkowski test, ACC deaminase activity measurement, biofilm production measurement, and GC-MS analysis of volatile compounds. *In vivo* assays using plant trials and plant-bacteria dual growth experiments were also carried out.

Indole-3-acetic acid (IAA), also known as auxin, governs different stages of plant growth and development such as cell elongation, cell division, and tissue differentiation, and influences apical dominance (Anwar et al., 2016). The IAA produced by rhizobacteria have effects on root systems by increasing root weight and size, branch numbers, and surface area (Patten and Glick, 1996; Glick, 2014; Goswami et al., 2016). Although there are several biosynthetic pathways (indole-3-acetanimide, indole-3-pyruvic acid, and indole-3-acetonitrile) for IAA synthesis, we focused on genes involved in tryptophan biosynthesis because IAA is primarily synthesized from tryptophan. A study done by Hu et al. (1999) showed that the expression of the genes *trpABC* was regulated by both the growth rate and phase. Hence, in this study, we harvested the cells of the strains at late-log phase (Figure 2A). Our transcriptome results showed that *trpABC* were upregulated in the mutants despite their lower growth rate compared with the wild-type. We also found a downregulation of the trp repressor which is involved in controlling biosynthesis of amino acids. The organization of *trp* genes within operons and regulatory mechanisms used to control *trp* operon expression greatly varies which reflects divergence in relationship to different metabolic contexts of tryptophan biosynthesis (Merino et al., 2008). Yanofsky (1984) states that when a monofunctional *trpG* is associated with other trp genes, it may exist as a separate gene, as seen in *S. marcescens* wherein it is adjacent with *trpD*. In a similar manner, the *trpG* and *trpD* found in GS2 are adjacent. This may explain why these two genes involved in tryptophan biosynthesis are the only ones which were down regulated result in contrast with *trpABCE.* It is perhaps that various arrangements of *trp* genes exist in microorganisms which provides an alternative to synthesize tryptophan (Yanofsky, 1984). Nonetheless, our *in vitro* assay for indolic compounds using the Salkowski test revealed a significant decrease in IAA production by the mutants in comparison to GS2 (Figure 2B). Moreover, *in vivo* plant assays showed a decrease in root lengths of plants treated with the mutant strains, as compared to the wild-type, GS2 (Table 1). To the best of our knowledge, this study reports, for the first time, the deletion of the autoinducer and the receptor genes affected the regulation of tryptophan biosynthesis genes.

The compound ACC, a direct precursor of ethylene, is degraded by ACC deaminase, thereby creating a concentration gradient that favors its exudation. Ethylene and auxin are related growth regulators, and maintaining a balance between them is important, as some effects attributed to auxin-producing bacteria are a result of ACC degradation (Glick, 2014; Goswami et al., 2016). In our study, we identified a gene coding for *D*-cysteine desulfhydrase, which is a homolog of ACC deaminase and belongs to the same family (Nascimento et al., 2014). The protein-BLAST search also confirmed that the ACC deaminase gene is present in GS2 genome and the ACC deaminase gene is located inside the *D*-cysteine desulfhydrase domain. Interestingly, our transcriptome analysis showed that this gene was down regulated in AI with a minute fold change in RT. Indeed, based on ACC deaminase activity assay, the amount of α-ketobutyrate per milligram of protein produced in the mutant strains was lower compared to GS2 (Figure 2C). Thus, the significant root length reduction of rice plants inoculated with AI and RT mutant strains compared to plants inoculated with GS2 indicates the role of QS genes in plant growth promotion via ACC deaminase regulation. This complies with the previous study (Danish and Zafar-ul-Hye, 2019) that ACC deaminase hydrolyses ACC exuded from plant roots and resulted in better root growth.

Biofilms are masses of microbial cells encased in a self-made matrix of extracellular polymeric substances (Valen and Scheie, 2018). Cells of PGPR, when present in a biofilm, should perform well because of inhibition of competing organisms, nutrient uptake, and improved adaptation to changing environmental conditions (Seneviratne et al., 2010; Backer et al., 2018). Biofilm production is known to be directly regulated by QS genes (Li et al., 2015; Kumari et al., 2016). There are two phases in motility-biofilm transition, the first being a fast inhibition at the level of flagellar function, second, a slow inhibition at the level of inactivation of flagellar gene expression (Guttenplan and Kearns, 2013). In this study, the cells were harvested and extracted only after 20 h of incubation, the late -log phase in which more active metabolism is found (Rossetti et al., 2009). In spite of this active phase, the deletion of the QS genes still affected the expression levels of the genes involved in biofilm formation. We identified that 46 genes present in GS2 are involved both in biofilm formation and motility. *FlhDC*, which is a master regulator for flagellar gene expression, is upregulated in RT while downregulated in AI, we still observed downregulation in most of the genes involved in flagellar formation. Of the 46 genes, more than half of them were downregulated in both mutants. Chemotaxis protein CheY, which controls the flagellar switch complex that determines the direction of flagellar rotation (Paul et al., 2011), was downregulated in both AI and RT mutants. In our *in vitro* assay, crystal violet staining was used to measure biofilm formation, and the results showed that the WT was able to form biofilms while the mutants failed to do so after 48 h of incubation. This confirms the contribution of QS genes to biofilm formation.

Bacteria produce a wide range of volatile compounds that have beneficial effect for the growth of plants (Chung et al., 2016; Sharifi and Ryu, 2018). Thus, we checked the role of QS genes for the production of volatile compounds, which aid in plant growth promotion. Here, we focus on acetoin which is known to have effects on plant growth and induction of systemic resistance in plants (Khalifa et al., 2016; Yi et al., 2018). Our transcriptome result showed that the genes responsible for acetoin production including *aldB, budB, ilyB* genes were minutely regulated in both mutants but *ilyH* was found to be downregulated. Similar to our findings, previous studies have reported the role of QS in acetoin production (Moons et al., 2011; Jin et al., 2019). In our qualitative *in vitro* assay, all strains were positive for acetoin production, however, our quantitative GC-MS data revealed that acetoin production in the mutants were less in comparison to the WT (Table 2). Similar to our results, Van Houdt et al. (2006) reported that knocked out QS signal production caused a shift toward reduced acetoin production in two *Serratia* species. In dual-growth experiments, we observed that most of the plant did not grow well, especially in higher inoculum concentrations, particularly in the LB media. This is likely due to plant growth inhibitory volatile compounds emitted from the strains. Blom et al. (2011) suggests that cyanide compounds emitted from bacteria may be involved in the plant growth inhibition. Interestingly, the results of our GC-MS data reinforces the dose response results. We detected cyanide compounds in both media and high concentration of cyanide was emitted from LB media (Methyl-o-cyanobenzoate and Phenylcyanamide) (Table 2).

We also theorize that there is perhaps an indirect effect of QS genes on the regulation of PGP activities. To this end, we considered the QS effects on carbon and nitrogen metabolism. Our transcriptome data showed that there were down regulation of a number of genes involved in the TCA cycle in both the AI and RT. Of the 17 genes we identified, six of them were downregulated in the receptor-deleted mutant while 11 genes were down regulated in the autoinducer-deleted mutant. Succinyl-CoA ligase, Isocitrate dehydrogenase, and Malate dehydrogenase genes were among the down regulated genes in both mutants. The cell densities of the mutant strains in liquid culture were observed to be lower than that of the WT strain during the transition to stationary phase. This finding is similar to the previous study (Lazazzera, 2000) that QS may play a more central role, wherein the QS pathways converge with starvation-sensing pathways to regulate cell transition into stationary phase. In addition, Davenport et al. (2015) reported that QS mutant altered the concentrations of the TCA cycle intermediates which lead to a readjustment in central metabolism. Furthermore, Wagner et al. (2003) identified 44 QS-regulated genes involved in nitrogen metabolism, using microarray analysis. Among the different nitrogen sources, ammonium is preferred because it supports more rapid growth (De Reuse and Skouloubris, 2001; Wang et al., 2017). Hence, we focused on the genes involved in ammonium synthesis. Subsequently, we identified a total of 16 genes associated with ammonium synthesis, of which 13 were down regulated in the autoinducer-deleted mutant, while only 5 genes were down regulated in the receptor-deleted mutant. An and his associates stated that QS may also control nutrient acquisition and help maintain homeostatic primary metabolism (An et al., 2014). Production of phytohormones are influenced by growth stage and availability of substrate (Priyadharsini and Muthukumar, 2017). Thus, down regulation of a significant number of genes involved in carbon and nitrogen metabolism could likely affect the production of IAA and ACC deaminase.

While our study showed the effect of the potential PGP traits and the QS activity *in vitro*, the effect remains to be observed in real soil conditions. Thus, further investigation on the effect of GS2 under real soil rhizosphere is needed in order to determine the production of the QS compounds and PGP potential. Since the inoculation of the strain in the *in vivo* pot experiment was 10^8^ CFU/g soil, it is noteworthy that the inoculant could have an enormous impact on the microbial community, especially in sterilized soil, which in turn could also produce indirect effects in the plant growth (Jo et al., 2020). Hence, the effect of the inoculation of the strain in the soil microbiome remains to be seen.

As QS genes are considered as global regulators, we speculated that there might be a connection to PGP activities of *S. fonticola* GS2, a bacterial strain with known PGP and QS activities. The results of genomic, transcriptomic, and phenotypic analysis confirmed that the QS autoinducer and receptor genes did have an effect on the PGP activities of strain GS2. Overall, our results suggest that some PGP activities may be regulated by AHL QS system in *S. fonticola* GS2. Interest in the use of more agriculturally useful microbes has increased recently. Thus, our results would highly contribute to creating healthier agriculture products by advancing quorum sensing systems in plant growth promoting rhizobacteria.

## Data Availability Statement

The datasets generated for this study can be found in: https://www.ncbi.nlm.nih.gov/bioproject/?term=PRJNA637445, https://www.ncbi.nlm.nih.gov/bioproject/?term=PRJNA597248.

## Author Contributions

BJ and J-HS managed and conceptualized the project. BJ, JI, G-SP, S-JH, and J-HS designed the experiment and prepared the project strategy. BJ, HP, S-DC, and JI analyzed the data and performed statistical analysis. HP, JI, BJ, and S-DC analyzed and visualized the data. CP, YJ, HJ, and M-CK collected samples and were involved in curation of data. BJ, JI, and ST wrote the main manuscript. All authors reviewed, contributed to revisions, and accepted the final version of the manuscript.

## Conflict of Interest

BJ was employed by the company CJ Cheiljedang Corp., South Korea. G-SP was employed by the company Atogen Co., Ltd., South Korea. HJ was employed by the company COSMAX BTI, South Korea. The remaining authors declare that the research was conducted in the absence of any commercial or financial relationships that could be construed as a potential conflict of interest.

## Footnotes

1 ftp://ftp.ncbi.nlm.nih.gov/blast/db/

2 http://www.uniprot.org/

3 http://blast.ncbi.nlm.nih.gov/Blast.cgi

4 http://www.dnalink.com/korean/index.html
