# Supplementary figures and images for: Quorum Sensing System Affects the Plant Growth Promotion Traits of Serratia fonticola GS2
Source: Front Microbiol. 2020 Oct 30;11:536865. doi: 10.3389/fmicb.2020.536865 (PMC7720635; doi:10.3389/fmicb.2020.536865)

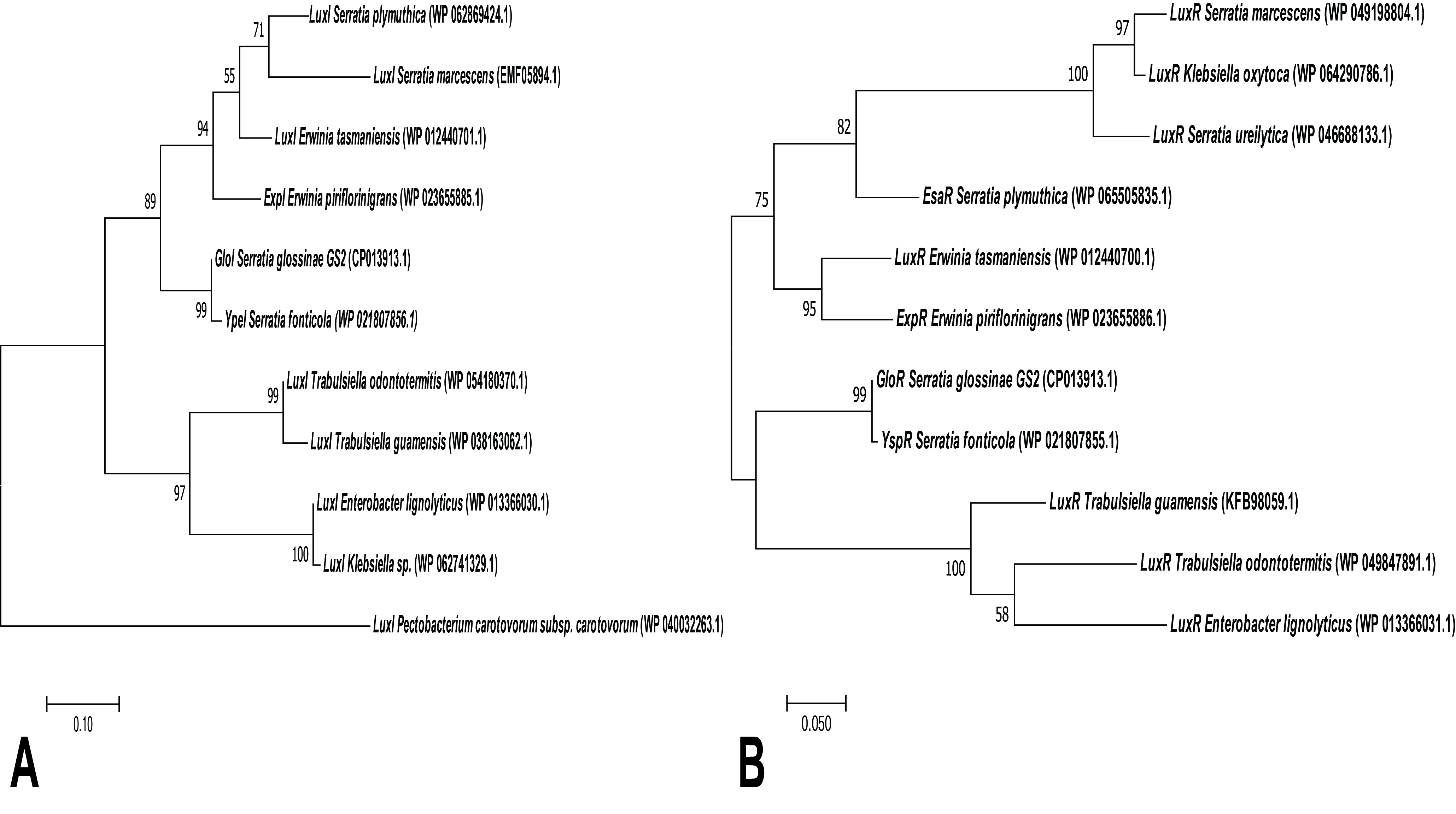

Supplement: Supplementary file 1 [file Image_1.TIF]

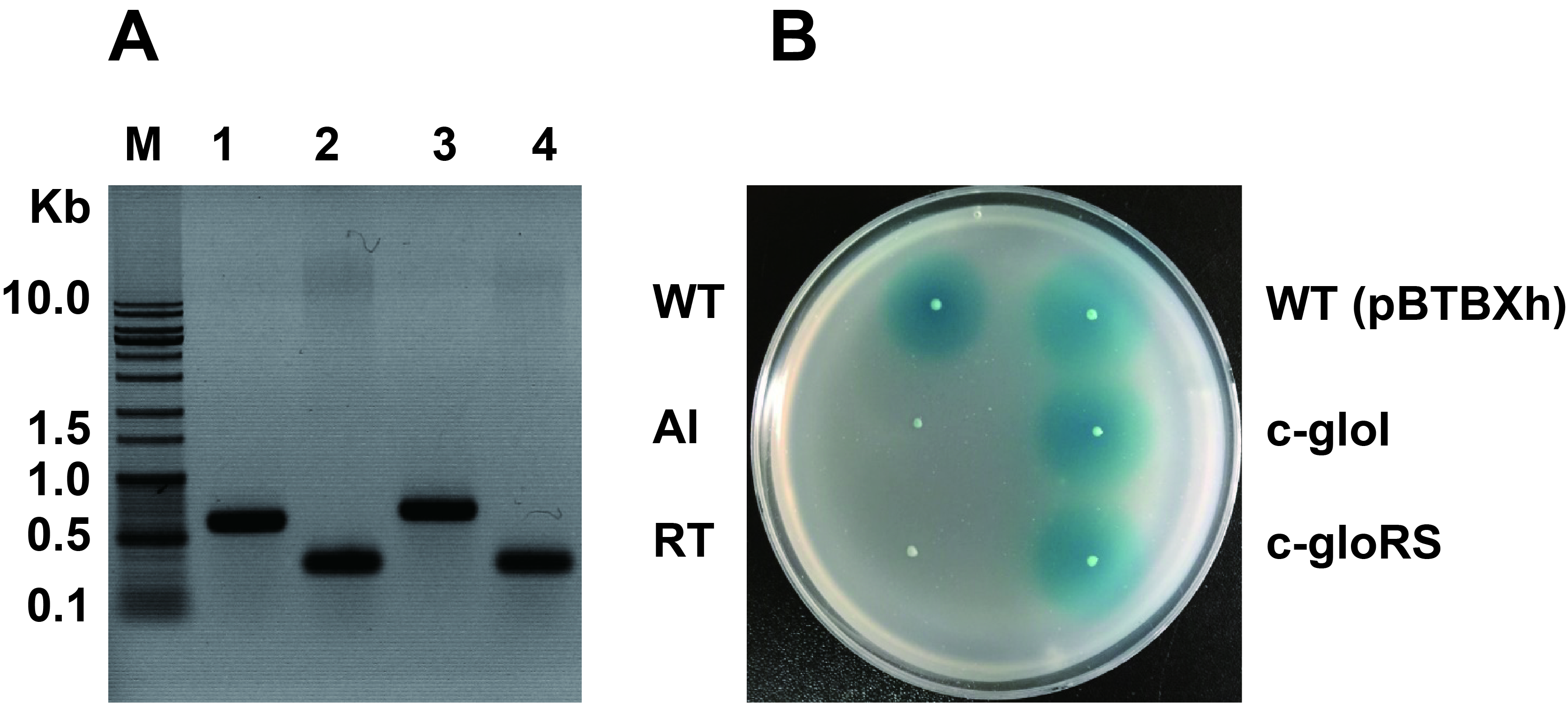

Supplement: Supplementary file 2 [file Image_2.TIF]

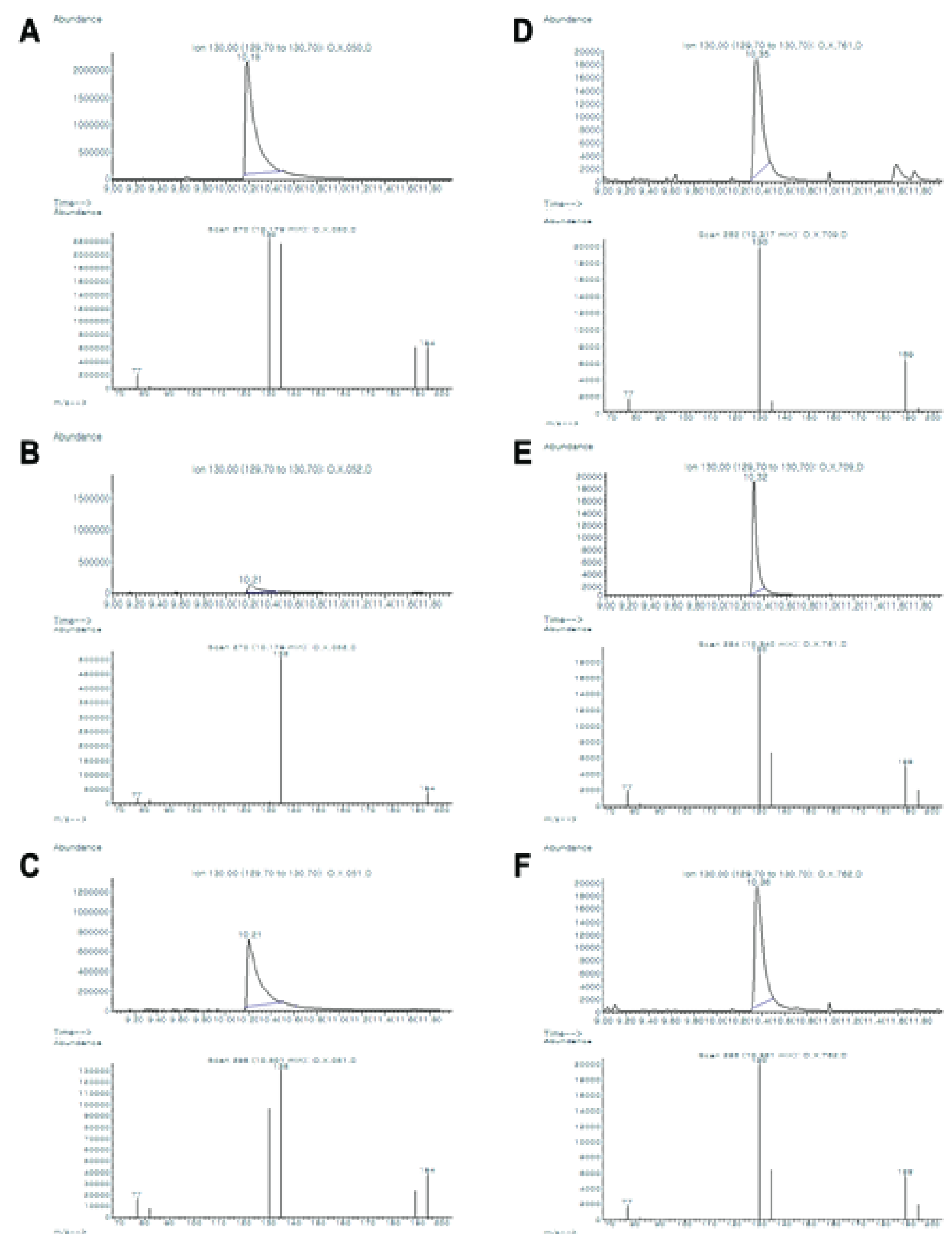

Supplement: Supplementary file 3 [file Image_3.TIF]

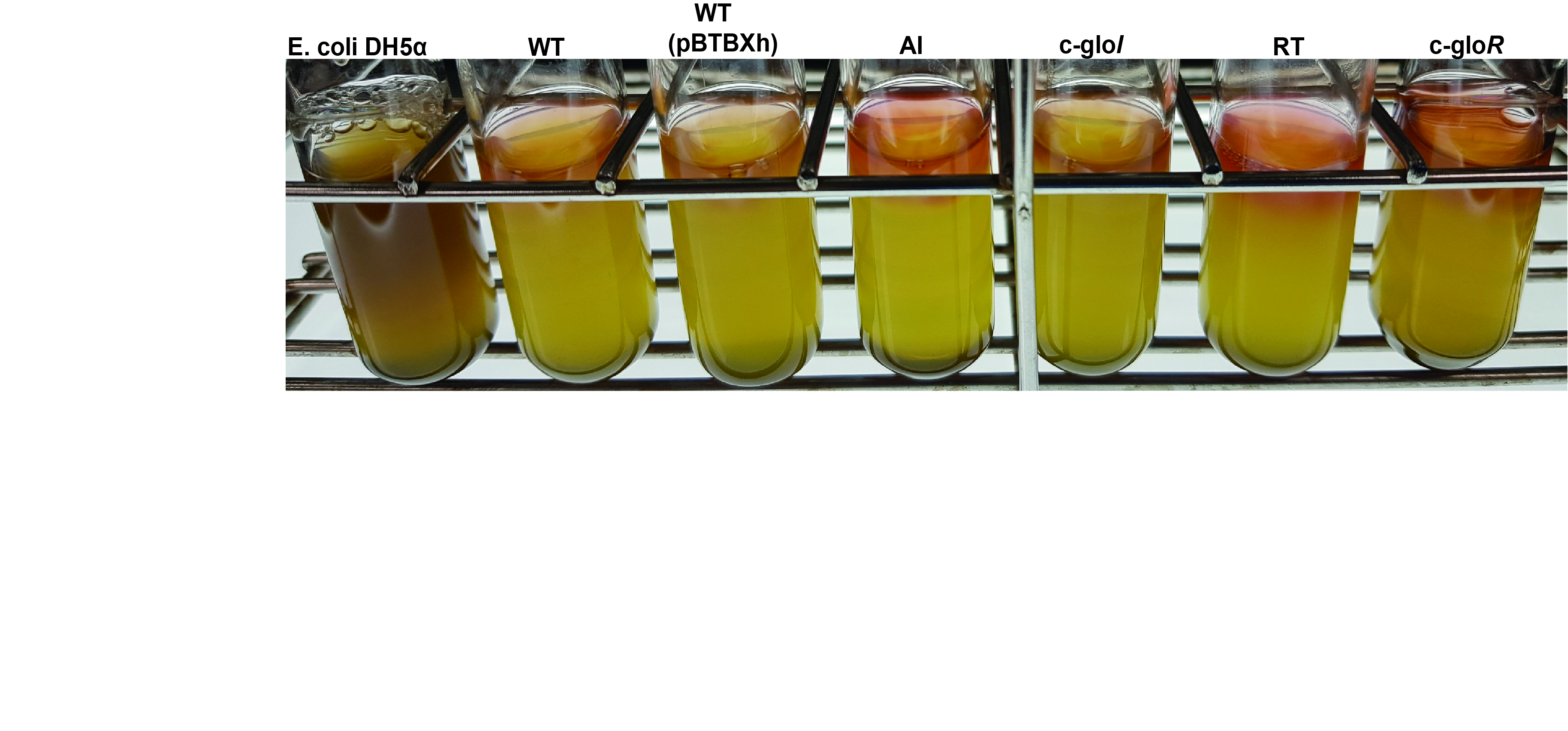

Supplement: Supplementary file 4 [file Image_4.TIF]

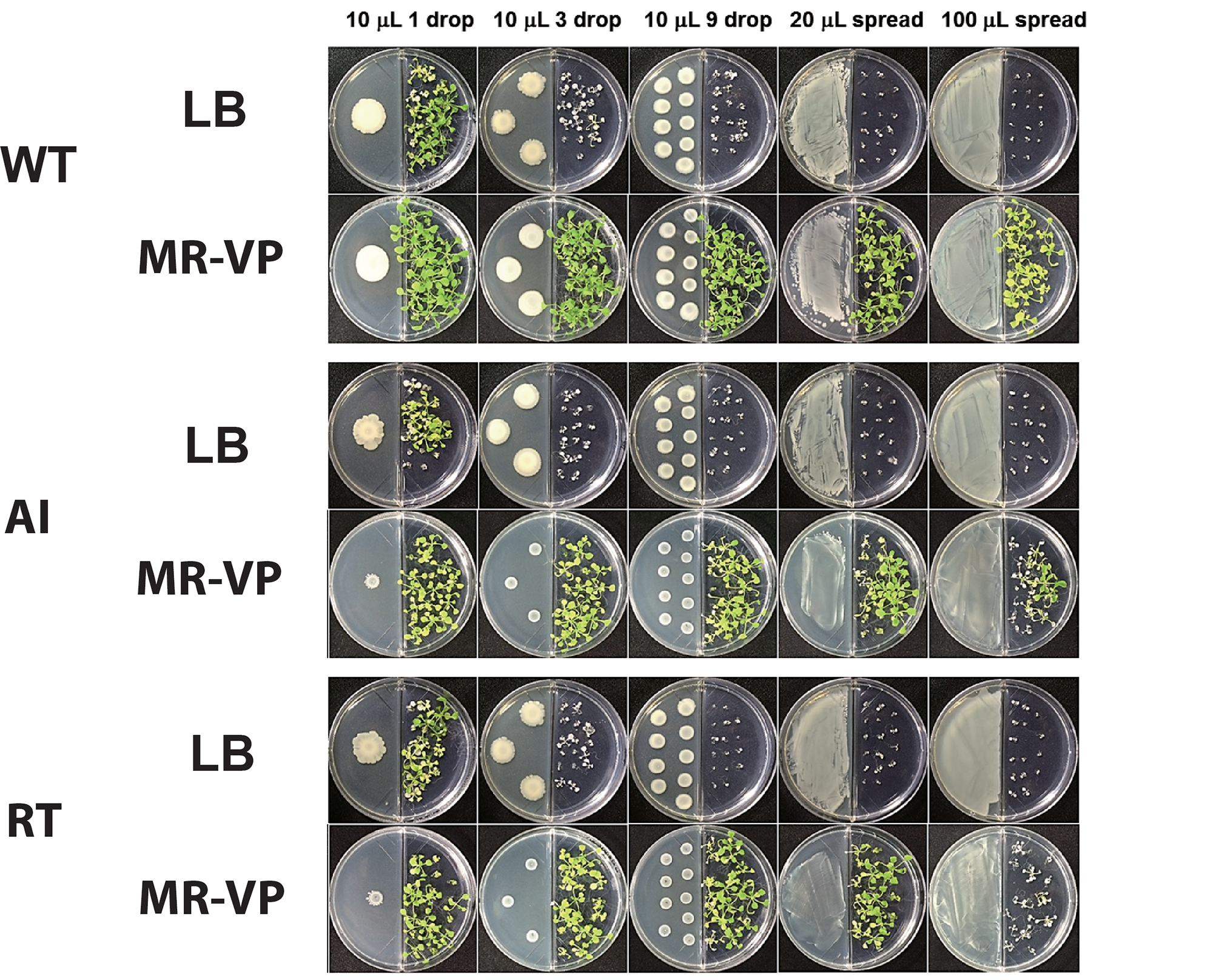

Supplement: Supplementary file 5 [file Image_5.TIF]
