# Supplementary material for: Quorum Sensing System Affects the Plant Growth Promotion Traits of Serratia fonticola GS2
Source: Front Microbiol. 2020 Oct 30;11:536865. doi: 10.3389/fmicb.2020.536865 (PMC7720635; doi:10.3389/fmicb.2020.536865)
Supplement: Supplementary file 6 [file Table_1.DOCX]

Supplementary Table 1. Primers used in this study

| **Primer** | **Sequence (5’ to 3’)*** |
| --- | --- |
| pDS132-specific-F | GATCGATCCTCTAGAGTCGACCT |
| pDS132-specific-R | ACATGTGGAATTGTGAGCGG |
| gloIF | GCTAGCATGTTTAATATTTACAGCGTTAACTATGC |
| gloIR | GCTCTAGAGCTTACAAAGAAGGCTCAG |
| gloRF | GCTAGCATGGAAAACGAAGAACATATCAGTAACAT |
| gloRR | GCTCTAGAGCTCAACTAACCGGTTTAATCAGTTG |
| gloI37-1 | AACTGCAGAACCAATGCATTGGGCAATGAGAAATCTGAAGATCTCT |
| gloI188-3 | GCTCTAGAGCATCATGCCGTTCTCCACGCCGAAAAT |
| gloI618-2 | TATCGAGCTCTCCCCCAGTCGTTCAGCCTGGTTT |
| gloI469-4 | GCTCTAGAGCAATGTCAGCGTGTTGGAAACCGGTGTTTCC |
| gloR33-1 | AACTGCAGAACCAATGCATTGGAAAACGCACCTCGAAGCGAC |
| gloR183-3 | GCTCTAGAGCATACTGCAAAACGTTGGCTTTATAATGCTCTACCCACTCA |
| gloR684-2 | TATCGAGCTCTCCCCCTAGACGTATTGCATGC |
| gloR534-4 | GCTCTAGAGCAAACGAAATCCTCTACTGGGCCA |

* Sequences for restriction enzyme sites are underlined
