# Supplementary material for: Quorum Sensing System Affects the Plant Growth Promotion Traits of Serratia fonticola GS2
Source: Front Microbiol. 2020 Oct 30;11:536865. doi: 10.3389/fmicb.2020.536865 (PMC7720635; doi:10.3389/fmicb.2020.536865)
Supplement: Supplementary file 7 [file Table_2.DOCX]

**Supplementary Table 2.** Strains and plasmids

|  | **Description** | **Reference** |
| --- | --- | --- |
| ***Strains*** |  |  |
| ***Escherichia coli*** |  |  |
| DH5α | *endA1 glnV44 thi-1 recA1 relA1 gyrA96 deoR nupG purB20 φ80dlacZΔM15 Δ(lacZYA-argF)U169, hsdR17(r_K_^–^m_K_^+^), λ^–^* | Invitrogen |
| DH5α *λpir* | *supE44 ΔlacU169 (ϕ80 lacZΔM15) hsdR17 recA1 endA1 gyrA96 thi-1 relA1 λpir* | Simon et al. (1983) |
| SM10 *λpir* | *thi thr leu tonA lacY supE recA::RP4-2-Tc::Mu Km λpir* | Donnenberg et al. (1991) |
| BW20767 | *RP4-2(Km::Tn7,Tc::Mu-1) leu-163::IS10 ΔuidA3::pir+ recA1 endA1 thiE1 hsdR17 creC510* | Metcalf et al. (1996) |
| ***Agrobacterium tumefaciens*** |  |  |
| NT1 (pDC141E33) | Gm^R^; *N*-acyl homoserine lactone sensor strain | Val and Cronan (1998) |
| ***Serratia fonticola*** |  |  |
| GS2 WT | Wild-type | This study |
| GS2 *ΔgloI* | Markerless *gloI* deletion mutant derived from GS2 | This study |
| GS2 *ΔgloR* | Markerless *gloR* deletion mutant derived from GS2 | This study |
| GS2 WT (pBTBXh) | pBTBXh-3 transformed into strain GS2 | This study |
| GS2 *ΔgloI* (pBTBXh-gloI) | pBTBXh-gloI transformed into strain GS2 *gloI* | This study |
| GS2 *ΔgloR* (pBTBXh-gloR) | pBTBXh-gloR transformed into strain GS2 *gloR* | This study |
| ***Plasmids*** |  |  |
| pDS132 | *R6K ori mobRP4 cat sacB* | Philippe et al. (2004) |
| pDS132ΔgloI | Δ*gloI* cloned into pDS132 | This study |
| pDS132ΔgloR | Δ*gloR* cloned into pDS132 | This study |
| pTOP | Amp^R^, Km^R^; cloning vector | Enzynomics, Korea |
| pBTBXh-3 | Cm^R^; broad-host-range expression vector | Prior (2010) |
| pBTBXh-gloI | pBTBXh-3 with *gloI* cloned | This study |
| pBTBXh-gloR | pBTBXh-3 with *gloR* cloned | This study |

Antibiotic abbreviations used in this study: ampicillin (Amp), kanamycin (Km), chloramphenicol (Cm), gentamicin (Gm)
