# Supplementary material for: Quorum Sensing System Affects the Plant Growth Promotion Traits of Serratia fonticola GS2
Source: Front Microbiol. 2020 Oct 30;11:536865. doi: 10.3389/fmicb.2020.536865 (PMC7720635; doi:10.3389/fmicb.2020.536865)
Supplement: Supplementary file 8 [file Table_3.DOCX]

**Supplementary Table 3.** Effect of volatile organic compounds by *Serratia fonticola* strain GS2 wild-type and mutants on the growth of A. thaliana Col-0*.*

| Media | Strain | Plant fresh weight relative to control (%) | | |
| --- | --- | --- | --- | --- |
|  |  | Dose^1^ | | |
|  |  | 1 drop | 3 drops | 9 drops |
| LB | WT | 180.26 ± 26.79^a^ | 90.41 ± 8.86^a^ | 46.59 ± 8.88^a^ |
|  | AI | 115.91 ± 11.10^b^ | 45.74 ± 3.54^b^ | 31.53 ± 3.69^a^ |
|  | RT | 123.51 ± 8.80^b^ | 30.26 ± 4.13^b^ | 14.20 ± 1.25^b^ |
| MR-VP | WT | 279.94 ± 26.40^a^ | 341.36 ± 30.48^a^ | 222.38 ± 21.01^a^ |
|  | AI | 127.93 ± 11.04^b^ | 160.34 ± 14.27^b^ | 160.00 ± 19.83^b^ |
|  | RT | 138.97 ± 16.34^b^ | 169.14 ± 13.30^b^ | 169.91 ± 18.22^ab^ |

^1^one drop contains 10 µL of each strain at a concentration of 10^9^ CFU mL^-1^. Values are expressed as the means ± SE. Different letters in column for each dose in their respective media show significantly different according to Duncan’s multiple range post hoc tests (*p* < 0.05).
